# Urgent transcatheter aortic valve implantation in an all-comer population: a single-centre experience

**DOI:** 10.1186/s12872-021-02347-1

**Published:** 2021-11-19

**Authors:** Arpad Lux, Leo F. Veenstra, Suzanne Kats, Wim Dohmen, Jos G. Maessen, Arnoud W. J. van ’t Hof, Bart Maesen

**Affiliations:** 1 Department of Cardiology, Maastricht University Medical Center+, Maastricht, The Netherlands; 2 Department of Cardiothoracic Surgery, Maastricht University Medical Center+, Maastricht, The Netherlands; 3 Cardiovascular Reasearch Institute Maastricht University, Maastricht, The Netherlands; 4 Department of Cardiology, Zuyderland Medical Centrum, Heerlen, The Netherlands; 5 Business Information Management, Maastricht University Medical Center+, Maastricht, The Netherlands

**Keywords:** Transcatheter valve implantation, TAVI, Urgency, Hospitalisation

## Abstract

**Background:**

When compared with older reports of untreated symptomatic aortic valve stenosis (AoS), urgent transcatheter aortic valve implantation (u-TAVI) seems to improve mortality rates. We performed a single centre, retrospective cohort analysis to characterize our u-TAVI population and to identify potential predictors of worse outcomes.

**Methods:**

We performed a retrospective analysis of 631 consecutive TAVI patients between 2013 and 2018. Of these patients, 53 were categorized as u-TAVI. Data was collected from the local electronic database.

**Results:**

Urgent patients had more often a severely decreased left ventricular ejection fraction (LVEF < 30%) and increased creatinine levels (115.5 [88–147] vs 94.5 [78–116] mmol/l; *p* = 0.001). Urgent patients were hospitalised for 18 [10–28] days before and discharged 6 [4–9] days after the implantation. The incidence of peri-procedural complications and apical implantations was comparable among the study groups. Urgent patients had higher in-hospital (11.3% vs 3.1%; *p* = 0.011) and 1-year mortality rates (28.2% vs 8.5%, *p* < 0.001). An increased risk of one-year mortality was associated with urgency (HR 3.5; *p* < 0.001), apical access (HR 1.9; *p* = 0.016) and cerebrovascular complications (HR 4.3; *p* = 0.002). Within the urgent group, the length of pre-hospital admission was the only significant predictor of 1-year mortality (HR 1.037/day; *p* = 0.003).

**Conclusions:**

Compared to elective procedures, u-TAVI led to increased mortality and comparable complication rates. This detrimental effect is most likely related to the length of pre-procedural hospitalisation of urgent patients.

**Supplementary Information:**

The online version contains supplementary material available at 10.1186/s12872-021-02347-1.

## Background

Recent studies have proven that Transcatheter Aortic Valve Implantation (TAVI) is a viable treatment option for symptomatic aortic valve stenosis (AoS), regardless of surgical risk [1–4]. These results will without doubt broaden the range of TAVI indications and subsequently lead to an increase in the number of TAVI procedures. Increased availability of TAVI’s will affect decision making for special patient groups, such as unstable or vulnerable patients. For hemodynamically unstable patients, European and American guidelines suggest balloon aortic valvuloplasty as an emergent solution, a bridge to definitive treatment. But they do not give advice on urgent clinical scenarios when patients are not in a critical condition but are not advised to leave the hospital without aortic valve replacement [5–7]. Published data, however, imply that urgent TAVI, despite higher mortality rates is a reasonable option to treat decompensated severe aortic valve stenosis [8–12]. We hypothesized that proper pre-procedural stabilisation of vulnerable, urgent patients would result in mortality rates comparable with elective procedures. We aimed to identify key characteristics within this vulnerable patient group and to better understand the impact of urgency on survival.

## Methods

### Study design and setting

We performed a retrospective cohort study of consecutive femoral or apical transcatheter valve implantations between 01 January 2013 and 30 September 2018. The diagnosis of aortic stenosis was made by the referring physician and was validated by the local Heart-Team and TAVI experts. One patient with a documented off-label indication, aortic valve insufficiency, was excluded from the analysis. The assessment of coronary arteries, aortic valve, ascending and descending aorta and iliofemoral arteries were performed as requested by local heart team protocols. Both self-expandable and balloon-expandable bio-prostheses were used, device selection was based on pre-procedural CT scans and expert consensus.

We selected 1-year TAVI survival as our primary outcome. Short term-survival, defined as in-hospital and 30-day mortality were selected as secondary outcome measures. All-cause mortality was collected by our local business intelligence management. A direct link to the municipal personal records database allowed the registration of the actual date of death. Demographic information, medical history, laboratory- and echocardiographic parameters, procedural data and admission and discharge dates were collected from the local electronic documents. Cerebrovascular complications and post-procedural pacemaker implantations are continuously collected for the Dutch heart registry and were also available. Definitions can be found on the website of the Dutch Heart Registry (www.nederlandsehartregistratie.nl).

Two pre-defined populations were compared, elective and urgent patients. We used the definition of the Dutch Heart Registry to categorize patients as urgent. Patients who were not admitted for an elective procedure but for medical reasons and yet needed an intervention during the same hospitalisation were categorized as urgent. These patients couldn’t be discharged without a definitive procedure. Inclusion in the urgent group was not limited by a completed, ongoing or not-yet-started TAVI assessment. For the urgent group, we manually collected the symptoms and markers of acute cardiac events. Emergent procedures (e.g., cardiogenic shock) were not included in the analysis.

### Statistical analysis

Distribution of continuous variables was confirmed with the Kolmogorov-Smirnoff test. Continuous variables with normal distribution were presented as mean with standard deviations. Non-Gaussian variables were reported as median with 25 and 75 percentiles. For categorical variables, both absolute numbers and percentages were shown. Categorical variables were compared with the X^2^ test. Continuous variables were compared with the Mann–Whitney test, as necessitated by their distribution. Unadjusted 1-year survival and survival after hospital discharge was compared with Kaplan–Meier Survival analysis and log-rank test. Cox-regression analysis was performed to identify possible predictors of 1-year mortality. The proportionality of hazard assumptions was checked by comparing the log minus log curves. Binary logistic regression was used to study the predictors of in-hospital mortality. Variables for the multivariable analyses were identified with univariable analyses. Age and gender were always added to the multivariable analyses. Statistical analysis was performed with SPSS (IBM Corp. Released 2017. IBM SPSS Statistics for Windows, Version 25.0. Armonk, NY: IBM Corp). A *p*-value < 0.05 was considered statistically significant.

### Ethical considerations

Patients gave written informed consent for the procedure before undergoing a TAVI. The retrospective analysis of their data and the publication of the results was approved by the ethical committee at Maastricht University and Maastricht UMC + (METC azM/UM). Approval number: METC- 2019-15239. All methods were carried out in accordance with relevant guidelines and regulations in the declaration of Helsinki.

## Results

### Study population

During the study period, 631 patients underwent either transfemoral or trans-apical aortic valve implantations. We registered 53 patients who met the criteria of urgency. Patient characteristics are summarized in Table 1. Urgent patients had a lower BMI (26 [23–28] and 27 [24–30], *p* < 0.05), higher creatinine values (115.5 [88–147] vs 94.5 [78–116]; *p* = 0.001) and were more likely to have a severely decreased left ventricular ejection fraction (LVEF% < 30%, 30% and 4%, *p* < 0.001) and a history of cardiothoracic surgery (34% and 22%, *p* < 0.05). (Table 1.)

**Table 1:** Study population and procedural characteristics

	All patients	Elective patients	Urgent patients	*p*
Age (years)	80 [76–84]	80 [76–84]/79.7 ± 6.2	79 [73–85]	0.233
Male gender	47.7% (301/631)	46.9% (271/578)	56.6% (30/53)	0.197
BMI	**26.5 [24.1–29.5]**	**26.7 [24.1–29.8]**	**25.6 [23.3–27.9]**	**0.027**
DM	27.3% (172/631)	27% (155/578)	32% (17/53)	0.411
Prior stroke	9.4% (59/631)	9.2% (53/578)	11.3%	0.607
COPD	14.1% (89/631)	13.8%	17%	0.53
Prior Cardiac surgery	22.8% (144/631)	**21.8%**	**34%**	**0.043**
Valve surgery	7.8% (49/631)	7.3%	13.2%	0.122
LVEF < 30%	**6.0% (38/631)**	**3.8%**	**30.2%**	**0.001**
GFR < = 30 ml/min/1,73 m2	26.8% (169/631)	26.5%	30.2%	0.559
Creatinine (umol/l)	**96 [79–119]**	**94.5 [78–116]**	**115.5 [88–147]**	**0.001**
Logistic Euroscore	**11.3 [7.8–18.3]**	**11.9 [7.7–18.1]**	**17.0 [10.1–25.6]**	**0.001**
Euroscore II	**3.0 [1.8–4.9]**	**2.9 [1.7–4.5]**	**5.3 [3.4 -10.9]**	** < 0.001**
Apical access	24.6% (155/631)	23.8%	32.1%	0.184
Complications stroke	2.1% (13/631)	2.1%	1.9%	0.926
Renal failure	0.6% (4/631)	0.5%	1.9%	0.230
PM within 30 days	9.7% (61/598)	9.5%	11.3%	0.308
Pre-TAVI hospitalisation (days)	**1 [1–1]**	**1 [1–1]**	**18 [10–28]**	** < 0.001**
Post-TAVI hospitalisation (days)	**4 [3–6]**	**4 [3–6]**	**6 [4–9]**	** < 0.001**
In-hospital mortality	**3.8% (24/631)**	**3.1%**	**11.3%**	**0.003**
1-year mortality	**10.1% (64/631)**	**8.5%**	**28.3%**	**0.001**

Significant differences are in bold

Complication-stroke: symptomatic cerebrovascular event, which leads to registration in the local complication database. Complication-renal failure: a decline in kidney function, which was significant enough to be registered in the local complication database. Variables with a Gaussian distribution are shown as mean  ±  standard deviation and variables with a non-Gaussian distribution are shown with median and 25–75 percentiles

BMI, Body Mass Index; DM, diabetes mellitus; CVE, cerebrovascular event in the medical history; COPD, Chronic Obstructive Lung Disease; LVEF, Left ventricular ejection fraction; PM, pacemaker

### Preoperative risk evaluation and hospitalisation

Urgent patients had a higher surgical risk (logistic Euroscore: 17 [10–26] vs. 11 [8–18]; Euroscore II: 5.3 [3.4–10.9] vs. 2.9 [1.7–4.5]; *p* < 0.001 for both). Elective patients were usually admitted one day before the implantation, while urgent patients had a median pre-procedural hospitalisation of 18 [10–28] days (*p* < 0.001).

### Procedural characteristics and complications

The frequency of apical access was equal in both study groups (urgent 32% vs. elective 24%, *p* > 0.05). Post-procedural hospitalisation was longer after urgent implantations (6 [4–9] vs. 4 [3–6] days, *p* < 0.001). Prevalence of post-procedural complications was comparable between the urgent and the elective groups (Table 1.)

### Survival and predictors of outcome

**Fig. 1:**
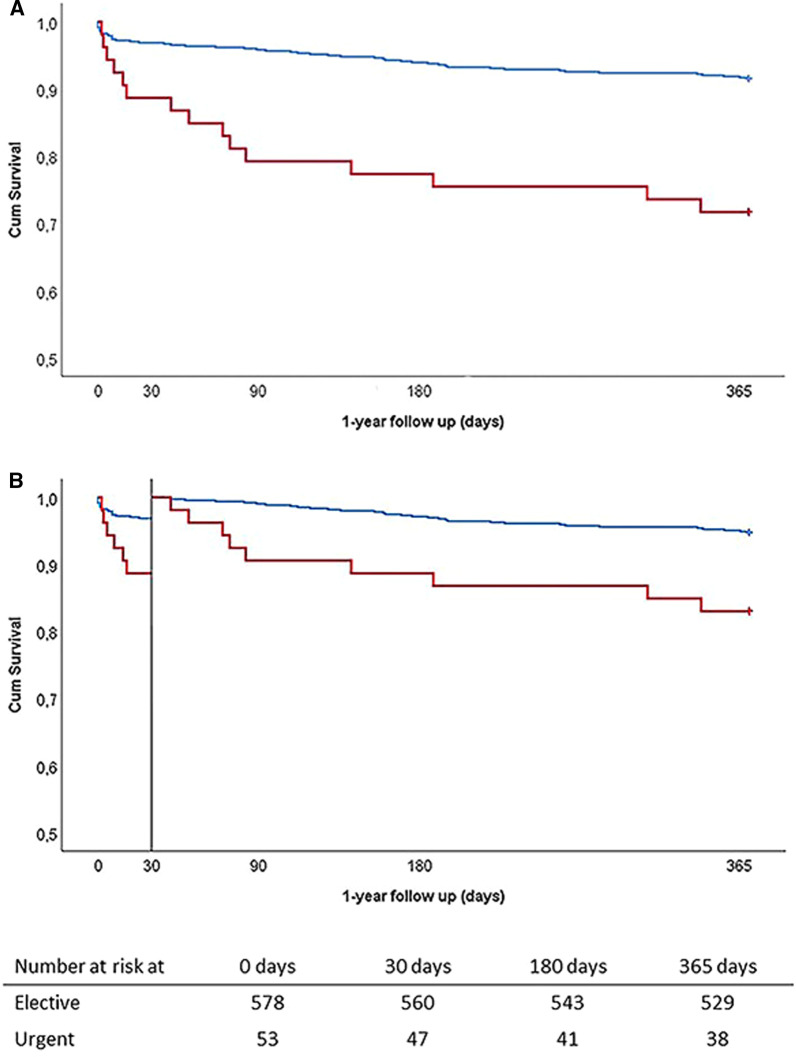
Post-TAVI survival (Kaplan–Meier curves). Panel **A** shows the cumulative 1- year survival after TAVI implantation. Panel **B** shows the landmark analysis with the landmark set at 30 days. Patients at risk are shown in the life table under panel **B**. Red = urgent patients; Blue = Elective patients

At 1-year follow-up, mortality was higher in the urgent group compared to the elective group for the whole cohort (28.3% vs 8.5% respectively, *p* < 0.001; Fig. 1.) and for the apical (41.2% vs 13.0%, *p* < 0.008) and femoral implantations (20.5% vs 7.0%, *p* < 0.005) as well. After identifying variables with a significant effect on mortality of the whole cohort (univariable analysis, listed in Table 2.), a multivariable analysis was performed. Urgency (HR 3.4, 95% CI 1.919–6.192; *p* < 0.001), apical access (HR 1.9, 95% CI 1.123–3.155; *p* = 0.045) and cerebrovascular complications (HR 4.3, 95% CI 1.685–11.212; *p* = 0.002) proved to be independent predictors for mortality at 1 year. To avoid redundancy, Euroscore II was not included in the multivariable model. (Table 2.) Repeating the same analysis for patients with femoral access only, confirmed the significant influence of urgency (HR 3.5 [1.6–7.5]; *p* = 0.002) on 1-year survival and showed that chronic lung disease (HR 2.3 [1.1–4.7]; *p* = 0.025) played an important role as well.

**Table 2:** Uni- and multivariable Cox regression analysis

	Hazard ratio	CI 95%	*p*-value	Hazard ratio	CI 95%	*p*-value
*Patient characteristics*						
Age	1.006	0.967–1.048	0.761	1.008	0.967–1.051	0.699
Gender	0.968	0.5931–1.581	0.897	0.993	0.595–1.657	0.978
Height	0.981	0.954–1.010	0.196			
Weight	0.984	0.966–1.003	0.093			
BMI	0.968	0.914–1.025	0.267			
DM	0.877	0.498–1.544	0.649			
Prior stroke	0.299	0.073–1.223	0.093			
COPD	1.551	0.844–2.852	0.158			
Creatinine	1.002	0.998–1.006	0.361			
GFR	0.989	0.975–1.003	0.111			
GFR < = 30	0.859	0.502–1.468	0.577			
LVEF < = 30	2.109	0.962–4.624	0.062			
Cardiac surgery	0.866	0.471–1.591	0.642			
*Hospitalisation and procedural risk*						
Euroscore II	**1.075**	**1.030–1.122**	**0.001**			
Urgency	**3.771**	**2.114–6.727**	** < 0.0001**	**3.447**	**1.919–6.192**	**0.001**
Apical access	**2.086**	**1.262–3.447**	**0.004**	**1.882**	**1.123–3.155**	**0.016**
Days to discharge*	1.055	1.030–1.081	< 0.0001			
*Complications*						
Major stroke	**4.815**	**1.931–12.007**	**0.001**	**4.346**	**1.685–11.212**	**0.002**
Renal failure	**6.909**	**1.688–28.283**	**0.007**	4.055	0.960–17.133	0.057
Pacemaker implantation	1.359	0.531–3.482	0.522			

Significant differences are in bold

Complication-stroke: symptomatic cerebrovascular event, which leads to registration in the local complication database. Complication-renal failure: a decline in kidney function, which was significant enough to be registered in the local complication database

BMI, Body Mass Index; DM, diabetes mellitus; CVE, cerebrovascular event in the medical history; COPD, Chronic Obstructive Lung Disease; LVEF, Left ventricular ejection fraction; PM, pacemaker

*Days from TAVI to discharge

Urgent patients had higher short-term mortality rates too (in-hospital and 30-day both 11.3% vs 3.1%; *p* = 0.011). (Fig. 1.) Patients who were discharged from the hospital, had lower perioperative risk, were younger with a higher BMI and a better LVEF. Apical access and periprocedural stroke were less frequently seen in the discharged group (Additional file 1: Table S1).

Even after hospital discharge, urgent patients retained a significantly higher mortality (19.1% vs 5.5%, Log-Rank *p* < 0.01). (Fig. 1.) Those who died within the first year were also more likely to have COPD and were discharged after a prolonged hospitalisation (Additional file 2: Table S2). After adding these variables to an age and gender corrected Cox-regression analysis, only urgency (HR 2.8, CI 1.3–6.1; *p* = 0.012) and post-TAVI hospitalisation (HR 1.05 /day, CI 1.02–1.09, *p* = 0.001) showed a significant interaction with the outcome.

### Symptoms and biomarkers of the urgent group

Out of the 53 patients, 16 (30.5%) had angina, 39 had symptoms of heart failure (73.6%) and 12 (22.6%) suffered from syncope at the moment of their hospital admission. As for the elective patients, the main presentation was dyspnoea (51% [294/578]) while there was a comparable prevalence of angina (35% [38/119]) with the urgent group. They had a median LVEF of 51 [30–60] % and a mean creatinine level of 122 ± 44 umol/l and a mean haemoglobin level of 7.1 ± 1.38 g/dl. Without a standardized admission protocol, only 38 (71%) patients had BNP measurements (787 [450–2078]) and 44 (83%) patients had creatine-kinase measurements (67 [46–110] mmol/l) available. (Table 3) Thirty-three (62%) patients were not known to the heart team and only 7 (13%) were finished with their pre-TAVI work-up. Thirty-eight (72%) patients needed a CT scan, 18 (34%) a coronary angiogram, 18 (34%) an additional imaging modality and 23 (43%) an interdisciplinary consult. We found individual cases of severe gastrointestinal bleeding, presence of severe aortic valve regurgitation, infectious disease (urinary tract, gastrointestinal, pneumonia and endocarditis), extracorporeal life support with severe limb ischemia. One patient underwent a balloon valvuloplasty before the urgent TAVI.

**Table 3:** Symptoms and biomarker levels within the urgent group

*Symptoms*	
Angina	30.5% (16/53)
Heart failure symptoms	73.6% (39/53)
Syncope	22.6% (12/53)
*Admission labs*	
Haemoglobin (n = 53)	7.12–1.38 g/dl
Creatinine (n = 52)	122 ± 44 umol/l
Pro-BNP (n = 38)	787 [450–2078] ng/l
Creatine-kinase (n = 44)	67 [46–110] mmol/l

### The impact of pre-procedural hospitalization

Within the urgent population, the length of pre-hospital admission was the only significant predictor of 1-year mortality (HR 1.037/day; *p* = 0.003). Since a prolonged pre-procedural hospitalisation was unique to urgent patients, its effect was not tested on the whole cohort.

## Discussion

Our analysis shows that urgent TAVI implantations, despite pre-procedural stabilization, have higher mortality rates than elective procedures. The worse outcome could be partially related to prolonged pre-procedural hospitalisations.

In our patient cohort, urgency is a composite of patient vulnerability and clinical instability. This urgency should not be confused with emergent procedures, as there is a significant difference in the haemodynamic stability of these patients. While emergent and life-saving procedures should be performed immediately after decision making, urgent procedures are reserved for vulnerable patients who are not fit enough for standard waiting lists but are no candidates for emergent procedures either. Current European guidelines give advice on haemodynamically unstable patients (emergent and life-saving procedures) and patients with non-cardiac urgencies, but do not discuss the treatment options for this special urgent group [7]. But thanks to the latest technological advancements and large-scale randomised controlled trials (RCT) TAVI will probably be more often considered as a treatment option for vulnerable patients in need of urgent interventions as well [1–4]. A recent comparison of balloon Aortic Valvuloplasty (BAV) and TAVI for acute decompensated AoS found no mortality differences but observed an increase in in-hospital and peri-procedural adverse events [13]. Therefore, the authors suggest that TAVI could be considered as a primary therapy for urgent indications [13]. Unfortunately, there remains uncertainty about the correct definition of urgency, and there are no RCTs available on this topic. Available publications report favourable outcomes of urgent procedures [8, 9].

The age and gender distribution of our cohort are comparable to those of the large intermediate-risk TAVI trials and the published urgent cohorts [2, 3, 9–11]. The leading symptom was dyspnoea for both study groups, with angina being fairly common (30–35%) as well. Although common comorbidities and traditional risk factors are difficult to compare, in the present urgent cohort the prevalence of a severely decreased LVEF (≤ 30%) surely does not lag behind other publications. Only an analysis of patients in cardiogenic shock observed a higher ratio of patients with an LVEF ≤ 30% [9–11]. The most important difference relative to most published patient groups lies in the definition of urgency. Most workgroups publishing on urgent and emergent TAVI deal with patients suffering from symptomatic and/or acute heart failure only [8, 9, 11]. We have found only one publication with a comparable definition for urgency [10]. Both in our and in the other mixed cohort, heart failure symptoms are the leading cause of urgency (73% and > 90%), which is followed by angina pectoris [10]. Despite the above-mentioned differences in the definition of urgency and the inclusion of emergent procedures, the 30-day and 1-year mortality rate of 11% and 29% within our cohort remained in the range of previously published mortality rates (30-day: 7.4–33.3% 1-year: 19–40.7%) [9–12]. Importantly, the observed one-year mortality rate remains reasonably lower than previously published 1-year mortality rates (43–59%) of untreated symptomatic aortic valve stenosis [14, 15]. Our findings regarding potential predictors of mortality are also in line with previous publications, as urgency [10, 12], non-femoral access (apical in our case) [10] and post-procedural complications [11] were already reported to impact early and 1-year mortality rates. We have shown, however, that peri-operative and long-term risk estimations should look for distinct risk factors.

Our other surprising finding was the detrimental effect of prolonged pre-procedural hospitalisations on urgent TAVI survival. The prolongation resulted probably from the clinical status of our patients and the number of additional analyses needed, and it was one of the main reasons behind the higher mortality rates of our urgent populations. Although a longer out-patient waiting list is usually associated with poor outcomes [16], it has also been shown, that accelerated TAVI for urgent patients results in a counterintuitive increase in early mortality rates [12]. We expected a beneficial effect of pre-procedural stabilisation and hypothesized, that outcomes would only be influenced by traditional risk factors. There is, however, insufficient data available to fully unmask causality within our cohort. We believe that we observed the combined harmful effect of prolonged hospitalisations and a critical illness, a phenomenon well known to the intensive care specialists [17]. For symptomatic AoS patients a critical illness is given [15], and despite stabilised haemodynamics, their mobility and physical activity is further limited by the hospital environment. This could further decrease the functional status of AoS patients [18, 19]. And poor functional status has already been associated with poor TAVI outcomes [20]. This decrease in functional status could also explain the prolonged post-procedural hospitalisations within our urgent cohort [21].

Due to its overall safety and favourable effects on patient rehabilitation and mobility, TAVI remains a promising choice for the treatment of critically ill AoS patients. It is also clear, that in the light of other publications it can improve short and long-term AoS outcomes [9, 10, 15, 22]. Therefore, it could further decrease the applicability and meaningfulness of BAV in urgent situations [7]. To improve the outcome of urgent TAVI, it is essential to identify the factors with the most impact on short- and long-term patient survival. We have shown that a stable clinical status alone does not warrant the improvement of patient outcomes and that the dilution of urgent cohorts with non-heart failure patients does not change survival when compared with other publications [9, 10, 12]. It seems, however, that decisiveness and probably the physical condition of AoS patients (e.g. functional status, frailty) is crucial for a better TAVI survival [19, 20].

## Limitations

In this paper, we present single-centre data on short- and long-term outcomes of consecutive TAVI implantations. The diagnosis of urgency was based on the clinical opinion of the treating physician and not on a combination of inclusion or exclusion criteria. Comorbidities, anthropometric data, lab data and echocardiographic measurements were registered as part of the daily practice and not monitored. There was no structured 1-year follow up and 30-day complication rates are only available for patients who returned for their regular 30-day out-patient consultation. There is limited or no data available on the biomarkers and symptoms of the elective group. Due to the relatively low number of urgent patients, the regression analysis is less reliable. Pre-procedural functional status was not assessed by objective means.

## Conclusion

Compared to elective procedures, urgent TAVI has increased mortality and comparable in-hospital complication rates. It may also improve patient survival when compared to the clinical course of untreated symptomatic aortic valve stenosis patients in previous publications. Favourable outcomes seem to depend on proper timing and a good haemodynamic and functional status, but further analysis is needed to accurately identify potential therapeutic, nursing, and physical pre and rehabilitation targets.

## Supplementary Information

**Additional file 1**. Supplementary table 1: Characteristics of patients who died during the index hospitalisation.

**Additional file 2**. Supplementary file 2: Characteristics of patients discharged from the hospital.

## Data Availability

The datasets generated and/or analysed during the current study are not publicly available due to the restrictions from national and institutional regulations. Data are available from the corresponding author on reasonable request and only after the approval of the competent ethical committee.

## Abbreviations

AoS: Aortic valve stenosis
BMI: Body mass index
BNP: Brain natriuretic peptide
GFR: Glomerular filtration rate
HR: Hazard ratio
LVEF: Left ventricular ejection fraction
OR: Odds ratio
RCT: Randomized controlled trial
TAVI: Transcatheter aortic valve implantation
u-TAVI: Urgent transcatheter aortic valve implantation
