# Supplementary material for: Urgent transcatheter aortic valve implantation in an all-comer population: a single-centre experience
Source: BMC Cardiovasc Disord. 2021 Nov 19;21:550. doi: 10.1186/s12872-021-02347-1 (PMC8603591; doi:10.1186/s12872-021-02347-1)
Supplement: Supplementary file 1 — Additional file 1. Supplementary table 1: Characteristics of patients who died during the index hospitalisation. [file 12872_2021_2347_MOESM1_ESM.docx]

Supplementary Table 1. Characteristics of patients who died during the index hospitalisation.

|  | **In-hospital death** | **n** | **Discharged** | **n** | **p** |
| --- | --- | --- | --- | --- | --- |
| **Age (years)** | **84 [81-87]** | **24** | **80 [76-84]** | **607** | **0.005** |
| Male gender | 45.8% | 11 / 24 | 47.8% | 291 / 607 | 1.0 |
| **BMI** | **24.5 [21.8-26.9]** | **24** | **26.7 [24.1-29.7]** | **607** | **0.003** |
| DM | 16.7% | 4 / 24 | 27.7% | 168 / 607 | 0.349 |
| Prior stroke | 0 | 0 / 24 | 9.7% | 59 / 607 | - |
| COPD | 2.2% | 2 / 24 | 14.3% | 87 / 607 | 0.558 |
| Prior Cardiac surgery | 4.2% | 6 / 24 | 22.7% | 138 / 607 | 0.805 |
| Valve surgery | 2% | 1 / 24 | 7.9% | 48 / 607 | 1.0 |
| **LVEF <30%** | **20.8%** | **5 / 24** | **5.4%** | **33 / 607** | **0.011** |
| GFR<=30 ml/min/1,73 m2 | 29.2% | 7 / 24 | 26.7% | 162 / 607 | 0.815 |
| Creatinine (umol/l) | 103 [78-137] | 24 | 96 [79-117] | 606 | 0.379 |
| **Urgency** | **25%** | **6 / 24** | **7.7%** | **47 / 607** | **0.011** |
| **Euroscore II** | **4.94 [2.78-14.0]** | **24** | **3.01 [1.77-4.80]** | **607** | **0.002** |
| **Apical access** | **50%** | **12 / 24** | **23.6%** | **143 / 607** | **0.006** |
| Complications |  |  |  |  |  |
| **Stroke** | **12.5%** | **3 / 24** | **1.6%** | **10** | **0.011** |
| renal failure | 4.2% | 1/24 | 0.5% | 3 / 607 | 0.144 |
| PM within 30 days | 33.3% | 1/3 | 10.1% | 60 / 595 | 0.276 |

BMI = Body Mass Index; DM = diabetes mellitus; CVE = cerebrovascular event in the medical history; COPD = Chronic Obstructive Lung Disease; LVEF = Left ventricular ejection fraction; PM = pacemaker. Complication – stroke: symptomatic cerebrovascular event, which leads to registration in the local complication database. Complication – renal failure: a decline in kidney function, which was significant enough to be registered in the local complication database. Variables with a Gaussian distribution are shown as mean +- standard deviation and variables with a non-Gaussian distribution are shown with median and 25-75 percentiles.
