# Supplementary material for: Urgent transcatheter aortic valve implantation in an all-comer population: a single-centre experience
Source: BMC Cardiovasc Disord. 2021 Nov 19;21:550. doi: 10.1186/s12872-021-02347-1 (PMC8603591; doi:10.1186/s12872-021-02347-1)
Supplement: Supplementary file 2 — Additional file 2. Supplementary file 2: Characteristics of patients discharged from the hospital. [file 12872_2021_2347_MOESM2_ESM.docx]

Supplementary Table 2. Discharged patients.

|  | **Deceased before 1-year follow-up** | **n** | **Survivors** | **n** | **p** |
| --- | --- | --- | --- | --- | --- |
| Age (years) | 79.5 [74.3-83.0] | 40 | 80 [76.0-84.0] | 567 | 0.293 |
| Male gender | 50% | 20 / 40 | 52.4% | 297 / 567 | 0.870 |
| BMI | 27.9 [24.7-30.7] | 40 | 26.6 [24.1-29.4] | 567 | 0.196 |
| DM | 30.0% | 12 / 40 | 27.5% | 156 / 567 | 0.717 |
| Prior stroke | 5.0% | 2 / 40 | 10.1% | 57 / 567 | 0.413 |
| **COPD** | **27.5%** | **11 / 40** | **13.4%** | **76 / 567** | **0.032** |
| Prior Cardiac surgery | 17.5% | 7 / 40 | 23.1% | 131 / 567 | 0.558 |
| Valve surgery | 4.2% | 2 / 40 | 8.1% | 46 / 567 | 0.761 |
| LVEF <30% | 5.0% | 2/40 | 5.5% | 31 / 567 | 1.0 |
| GFR<=30 ml/min/1,73 m2 | 30.0% | 12 / 40 | 26.5% | 150 / 567 | 585 |
| Creatinine (umol/l) | 92.5 [80.3-138.5] | 40 | 96.0 [69.0-117.0] | 566 | 0.756 |
| **Urgency** | **19.1%** | **9 / 40** | **6.7%** | **38 / 567** | **0.002** |
| Euroscore II | 3.23 [1.77-5.45] | 40 | 2.99 [1.78-4.77] | 567 | 0.645 |
| Apical access | 32.5% | 13 / 40 | 22.9% | 130 / 567 | 0.179 |
| **Days to discharge** | **5 [3-10]** | **40** | **4 [3-6]** | **567** | **0.018** |
| Complications |  |  |  |  |  |
| Stroke | 5.0% | 2 / 40 | 1.4% | 8 / 567 | 0.136 |
| renal failure | 2.5% | 1 / 40 | 0.4% | 2 / 567 | 0.185 |
| PM within 30 days | 11.4% | 4 / 35 | 10.0% | 56 / 560 | 0.771 |

BMI = Body Mass Index; DM = diabetes mellitus; CVE = cerebrovascular event in the medical history; COPD = Chronic Obstructive Lung Disease; LVEF = Left ventricular ejection fraction; PM = pacemaker. Complication – stroke: symptomatic cerebrovascular event, which leads to registration in the local complication database. Complication – renal failure: a decline in kidney function, which was significant enough to be registered in the local complication database. Variables with a Gaussian distribution are shown as mean +- standard deviation and variables with a non-Gaussian distribution are shown with median and 25-75 percentiles.
